# New methods for confocal imaging of infection threads in crop and model legumes

**DOI:** 10.1186/s13007-021-00725-6

**Published:** 2021-03-07

**Authors:** Angus E. Rae, Vivien Rolland, Rosemary G. White, Ulrike Mathesius

**Affiliations:** 1 Department of Plant Sciences, Research School of Biology, Australian National University, Acton, ACT 2601 Australia; 2 CSIRO Agriculture and Food, GPO Box 1700, Acton, ACT 2601 Australia

**Keywords:** Cell wall, *Cicer arietinum*, Infection thread, Legumes, *Lotus japonicas*, *Medicago truncatula*, Nodulation, Rhizobia, Root hair, *Trifolium repens*

## Abstract

**Background:**

The formation of infection threads in the symbiotic infection of rhizobacteria in legumes is a unique, fascinating, and poorly understood process. Infection threads are tubes of cell wall material that transport rhizobacteria from root hair cells to developing nodules in host roots. They form in a type of reverse tip-growth from an inversion of the root hair cell wall, but the mechanism driving this growth is unknown, and the composition of the thread wall remains unclear. High resolution, 3-dimensional imaging of infection threads, and cell wall component specific labelling, would greatly aid in our understanding of the nature and development of these structures. To date, such imaging has not been done, with infection threads typically imaged by GFP-tagged rhizobia within them, or histochemically in thin sections.

**Results:**

We have developed new methods of imaging infection threads using novel and traditional cell wall fluorescent labels, and laser confocal scanning microscopy. We applied a new Periodic Acid Schiff (PAS) stain using rhodamine-123 to the labelling of whole cleared infected roots of *Medicago truncatula*; which allowed for imaging of infection threads in greater 3D detail than had previously been achieved. By the combination of the above method and a calcofluor-white counter-stain, we also succeeded in labelling infection threads and plant cell walls separately, and have potentially discovered a way in which the infection thread matrix can be visualized.

**Conclusions:**

Our methods have made the imaging and study of infection threads more effective and informative, and present exciting new opportunities for future research in the area.

**Supplementary Information:**

The online version contains supplementary material available at 10.1186/s13007-021-00725-6.

## Background

**Fig. 1:**
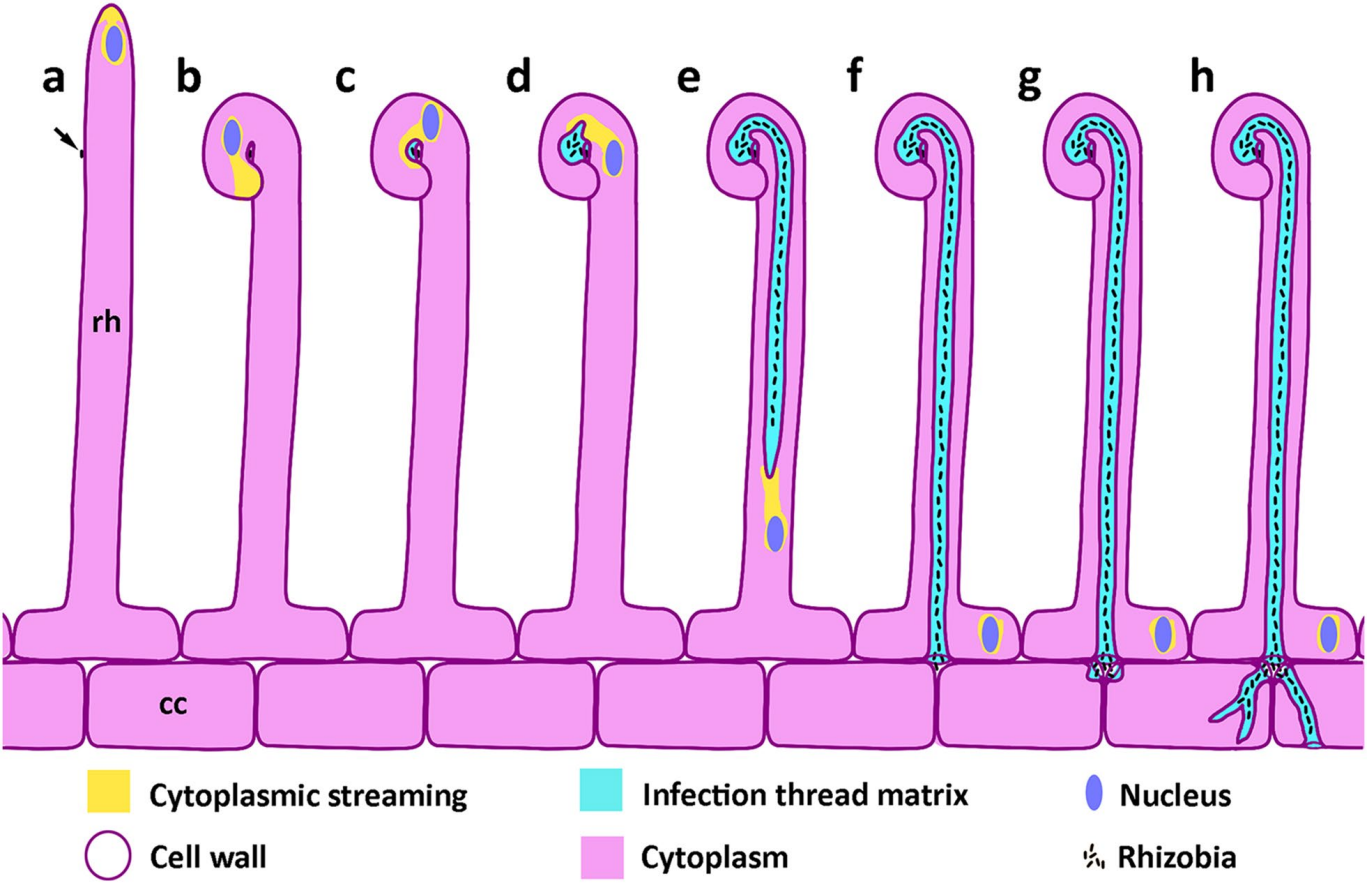
Infection thread formation. **a** A rhizobium cell (arrow) makes contact close to the growing tip of a root hair (rh). **b** Nod factors produced by the rhizobia trigger the tip of the root hair to curl around the bacterial cell, creating an enclosed pocket known as the infection chamber. **c** The infection chamber expands inwards as the bacteria begin to divide; exocytotic activity (yellow) at the root hair tip shifts to surround the chamber, secreting a matrix (cyan) into the infection chamber. **d** The infection chamber expands further as bacteria continue to divide, and the infection thread initiates by an inverted tip-growth from a localised weakening of the chamber wall. **e** The infection thread elongates through the root hair, preceded by the nucleus which maintains cytoplasmic streaming to the infection thread tip. **f** The infection thread fuses with the basal wall of the root hair, and bacteria are released into the intracellular space. **g** Localised areas of adjacent cortical cells are weakened and expand inwards to form new infection threads.** h** Cortical infection threads continue to elongate, often branching; and reforming in adjacent cells to carry rhizobia further into the developing nodule. Infection thread formation is varied within and between species, and the specific variants shown here—such as the ‘shepherds crook’ root hair curl, and the formation of two cortical threads from the junction of three cells—were chosen to best illustrate the main stages of formation. *rh* root hair, *cc* cortical cell

Most legume species form a symbiosis with rhizobia, which are nitrogen fixing bacteria. The rhizobia reside within root nodules, in which atmospheric nitrogen is fixed into a form that can be used by the host in exchange for fixed carbon. In most legumes, including the model legumes *Medicago truncatula* and *Lotus japonicus*, nodulation begins when a bacterial cell makes contact with the tip of a growing root hair cell, and induces the formation of an infection thread. This physical contact combined with Nod factors produced by compatible rhizobia triggers the tip of the root hair to curl around the bacterial cells, creating an enclosed pocket known as the infection chamber [1, 2]. In the infection chamber, the bacteria begin to divide, and exocytotic activity at the root hair tip shifts to surround the chamber [3]. The chamber expands as the bacteria divide, and as a matrix that presumably supports bacteria growth and cell wall remodelling is exuded into the chamber [4] (Fig. 1). The nature and complete function of the matrix is not well characterised, but it has been shown to contain proline-rich glycoproteins including ENOD11, nodule expansion proteins, and a nodule pectate lyase [3, 4].

The infection thread initiates from a weakened area of the infection chamber that extends into the root hair to form a tube [3]. Essentially, the bacteria remain inside the matrix, and thus outside the plant cell wall, the wall of the infection thread being continuous with the root hair cell wall, and containing similar cell wall components including cellulose, esterified and non-esterified pectin as well as xyloglucans [5]. The elongation of the infection thread is guided by reorganised microtubules [6, 7], and is preceded by the root hair nucleus, which is connected to the infection thread by a column of cytoplasmic material that moves through the cell in front of the thread [2] (Fig. 1). Exocytosis of cell wall material into the infection chamber and growing infection thread is crucial for its elongation, and this requires a complex composed of the exocyst unit EXO70H4, that interacts with vapyrin and the putative E3 ligase LUMPY INFECTIONS (LIN) [8].

The infection thread extends through the infected root hair where it fuses with the inner tangential wall of the root hair cell, releasing the bacteria into the intracellular space between the root hair and neighbouring cortical cell [2] (Fig. 1). Infection thread initiation and elongation continues through cortical cell layers until rhizobia are released into dividing cortical cells, where they differentiate into nitrogen-fixing symbiosomes, vesicles of plant membrane encapsulating one or more bacterial cells [9]. Prior to the formation of a new infection thread, the cortical cell nucleus moves towards the centre of the cell and a cytoplasmic bridge forms, analogous to phragmoplast formation in preparation for mitosis [5]. The growing infection thread then follows the path of this cytoplasmic bridge, which is aligned between adjacent cortical cells [2, 5]. Infection threads continue to be formed inside growing nodules. From the growing nodule primordium, infection threads grow and branch towards the nodule apical meristem to colonise new cells located between the nodule meristem and the infected zone of the nodule [10].

The mechanism of infection thread elongation has been compared to that of transfer cell wall ingrowths—which are localised deposits of cell wall material—coupled with a pulling effect by the cell cytoskeleton [11]. However, the infection thread was later confirmed to be a closed tube of cell wall [3], not an open-ended tube [12]. An alternative hypothesis is analogous to pollen tube tip-growth, with turgor pressure in the thread driving growth, but there is little experimental evidence supporting this hypothesis [1, 13]. Hardening of the matrix of the infection thread through cross-linking of glycoproteins containing arabinogalactan and extension subunits a short distance behind the tip of the infection thread has also been suggested as a mechanism of infection thread extension [11].

Current research aims to understand the legume/rhizobia symbiosis, to determine how the relationship first formed, and how it has adapted to its present form. The end-goal of this research is to reproduce the symbiosis in other significant crop species, and our limited understanding of infection thread formation is a significant barrier to reaching this goal. Entry via an infection thread is thought to be a more recent adaptation in nodulation, in contrast to the likely more ancestral infection via breaks in the root epidermis [14, 15]. Thus, an understanding of the genetics and mechanisms behind infection threads will contribute to our understanding of the evolutionary basis of this symbiosis.

A barrier to understanding infection thread development, especially their cell wall modification and extension during growth, is the lack of a method to visualise the three-dimensional structure of infection threads in sufficient detail. In most studies, infection threads are visualised indirectly using GFP-tagged rhizobia within threads [8, 16–19], which is convenient in that it requires no sample preparation. Simple histochemical staining can be used to show LacZ-tagged rhizobia with X-Gal, but this reveals little structural detail of infection threads. Bright-field imaging of threads containing GFP-labelled rhizobia shows the outline of the thread, but cannot generate 3D information, especially since young threads are enclosed in dense cytoplasm that obscures their structure [1]. Inside nodules, the presence of GFP-labelled bacteroids additionally obscures the network of infection threads.

Two other commonly used methods for imaging infection threads are Toluidine Blue staining of semi-thin embedded tissues and transmission electron microscopy for detailed ultrastructure [5, 20]. Obtaining 3D information by reconstructions from serial TEM sections has been explored [10]; however, lengthy sample prep and imaging time, sectioning artifacts, and human error in manual reconstruction are drawbacks to such an approach. An alternative approach is confocal imaging of infection threads labelled with fluorescent dyes. Confocal optical sectioning would enable 3D reconstructions of infection thread structure and achieve resolution in the tens of nanometres with super-resolution techniques. Moreover, the optical sectioning possible by confocal microscopy also allows infection threads to be resolved throughout whole nodules without mechanical sectioning. Despite these advantages, confocal imaging has not been fully utilised in this way, with only a few studies showing infection thread walls labelled with calcofluor white in thin sections [18, 21–23].

Of the many fluorescent dyes used for plant cell wall staining, the most effective for imaging in whole plant samples has proven to be variations on the Periodic Acid-Schiff (PAS) method [24, 25]. This method achieves uniform and stable labelling of plant cell walls by covalent binding of suitable Schiff-like dyes to aldehyde groups produced by oxidation of cell wall polymers by periodic acid. The dye used for this method has primarily been propidium iodide in Schiff solution but it was recently improved by the use of the brighter rhodamine-123, with the Schiff solution replaced by water [26]. We applied this newly improved PAS method to image infection threads in whole samples as it is an exceptionally bright and stable cell wall stain, and allows for deep optical sectioning (~ 400 µm in cleared, stained plant tissue).

## Results

**Fig. 2:**
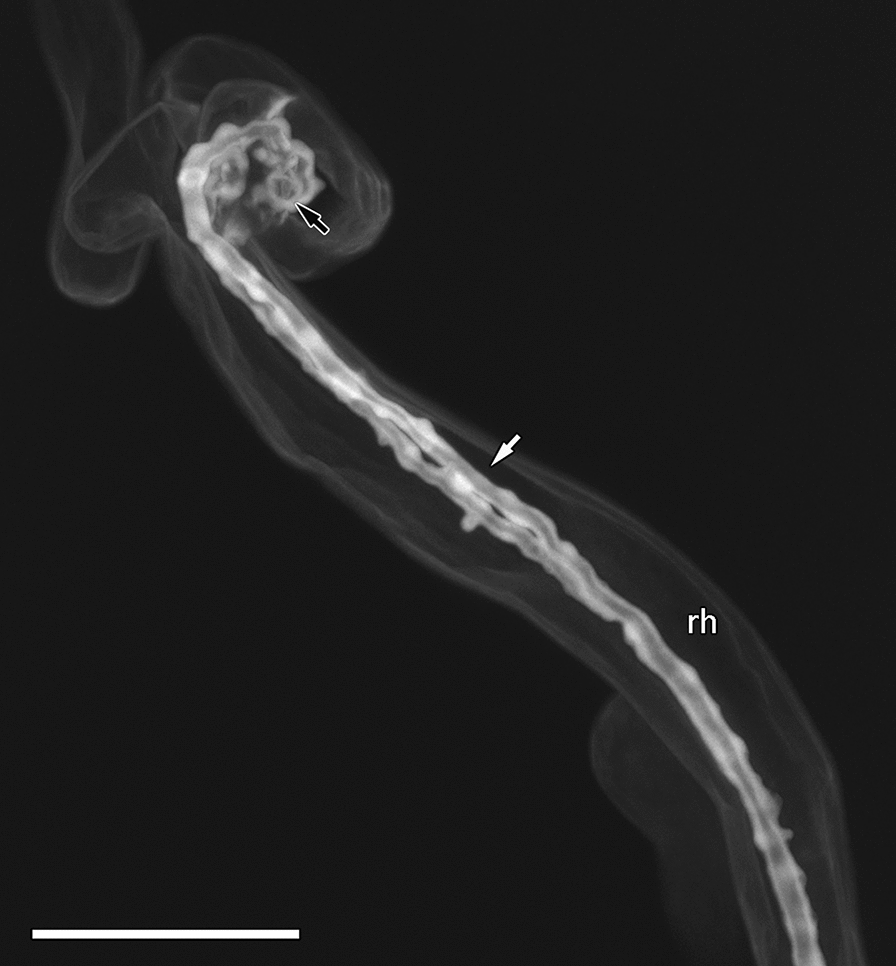
Infection thread after rhodamine-123 PAS labelling. Mature infection thread (white arrow) in *M. truncatula* root hair imaged with a 63× objective using a Leica SP8 confocal microscope. Maximum intensity z-projection from a 21.02 µm confocal z-stack. Scale bar equals 25 µm. Black arrow indicates position of infection chamber. *rh* root hair

To label infection threads with the rhodamine-123 PAS method, whole infected roots of *M. truncatula* were fixed, bleach-cleared to remove all cell components leaving only cell walls, oxidised with periodic acid, and stained with rhodamine-123. Stained samples were then mounted in glycerol and imaged by confocal microscopy. This allowed us to obtain much three-dimensional detail of the infection threads and the modified root hair containing them (Fig. 2). The image in Fig. 2 is a maximum projection of a confocal z-stack taken on a Leica SP8, and the infection thread is made visible by digital section ‘cutting’ the surface of the curled root hair cell to reveal the internal thread. In this example, there are two infection threads, and both are covered with small, sub-micrometre protuberances, giving it a ‘lumpy’ appearance. The infection chamber in this example is also visible at the infection thread origin (Fig. 2, black arrow).

This method revealed new three-dimensional details of infection threads and allowed us to investigate whether infection thread walls could be differentiated from the surrounding plant cell walls. Although infection thread walls are continuous with the walls of the cell they occupy, they do not autofluorescence with the same excitation [3]. This indicates a difference in infection thread wall composition that might be targeted. The compositional difference responsible for the lack of autofluorescence is not known, however there are many fluorescent dyes that label specific or unknown components of plant cell walls [27], and it is reasonable to hypothesise that one such dye might label infection threads more or less intensely than surrounding cell walls.

**Fig. 3:**
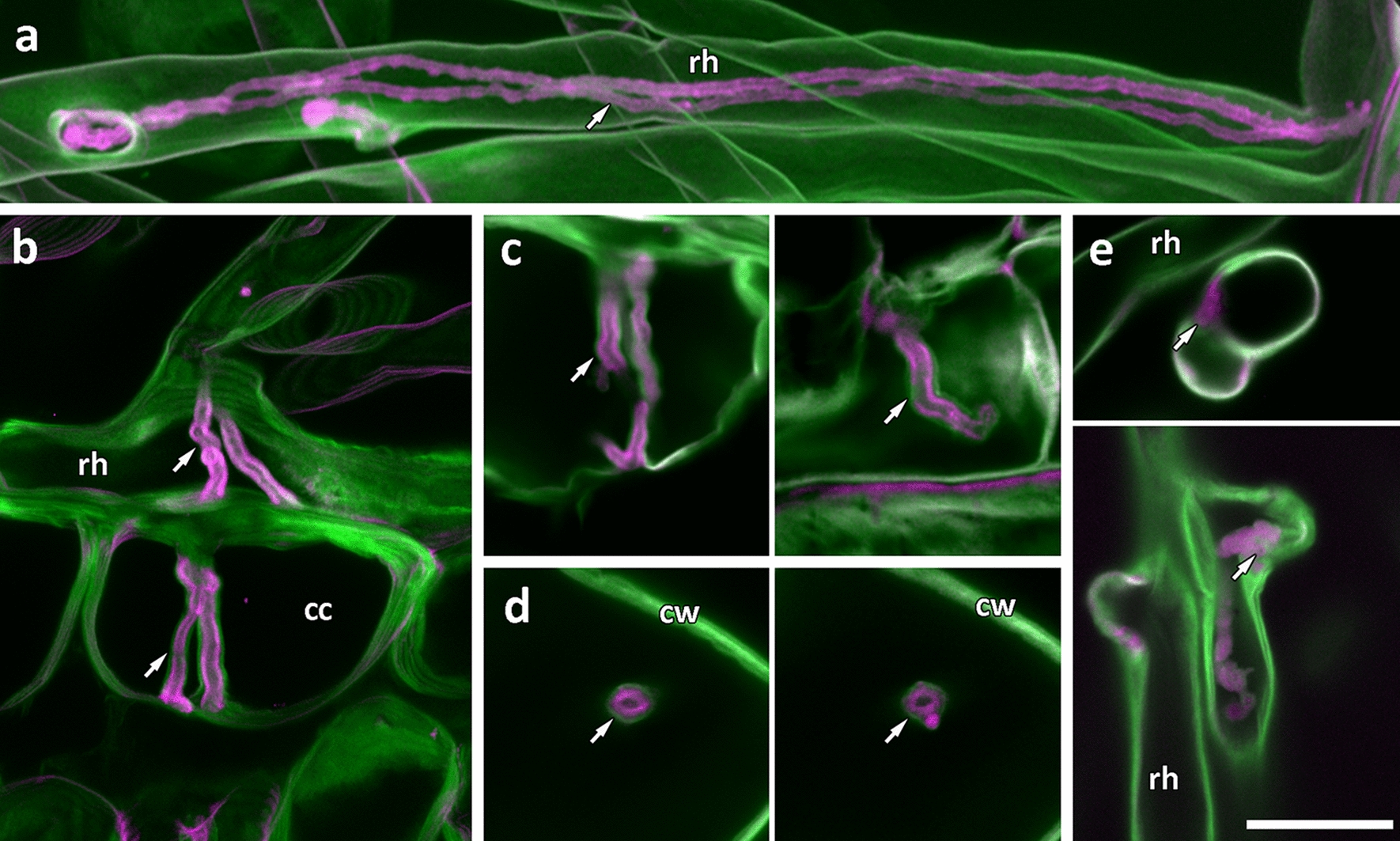
PAS rhodamine-123 counterstained with calcofluor white differentially labels, and shows distinct layers in, infection threads. *M. truncatula* root hairs stained with PAS rhodamine-123 (magenta) then with calcofluor white (green). **a** Root hair infection threads (arrow) inside a root hair are stained with rhodamine-123 whereas the dominant stain in cell walls is calcofluor white. **b**–**d** Cortical infection threads show two distinct layers, an outer layer stained with calcofluor white, and an inner layer stained with rhodamine-123. In (**b**) two infection threads exit the root hair to traverse the subtending epidermal cell and emerge in the cortical cells below. **c** Infection threads in root cortical cells. **d** Optical cross sections of infection threads in root cortical cells. **e** Infection chambers are also differentially labelled with rhodamine-123 (arrows). Images **a** and **b** are maximum projections of 60.01 µm, and 9.99 µm confocal z-stacks. All images were captured using a 63× objective. Scale bars = 25 μm. *rh* root hair, *cc* cortical cell, *cw* cell wall

We tested a range of fluorescent dyes that bind to plant cell walls, individually and in combination (see Additional file 1: Figure S1 for examples), and the most successful combination was that of rhodamine-123 with a calcofluor white counterstain. Infection threads were clearly delineated by the rhodamine-123 label in contrast to surrounding cell walls labelled with calcofluor white (Fig. 3). Infection threads were differentially labelled in this way in both root hairs (Fig. 3a) and as they extended into underlying cortical cells (Fig. 3b). Closer inspection revealed that the brightest rhodamine label was localised not to the entire infection thread wall, but to a thick inner layer, surrounded by a thin outer layer labelled more strongly with calcofluor white (Fig. 3b–d). This layering was most evident in cortical cell infection threads (Fig. 3c), and optical cross sections clearly show the two separate layers with an unstained compartment in the centre of the thread (Fig. 3d). Infection chambers also showed clear rhodamine-123 label before and after extension of an infection thread (Fig. 3e, white arrows). Three dimensional reconstructions of infection threads from confocal z-stacks made it possible to visualise how they develop within the root hair cell (Additional file 2: Movie S1, Additional file 3: Movie S2), as they emerge from root hair curls (Additional file 4: Movie S3), and in relation to other threads within single root hairs (Additional file 2: Movie S1, Additional file 4: Movie S3).

**Fig. 4:**
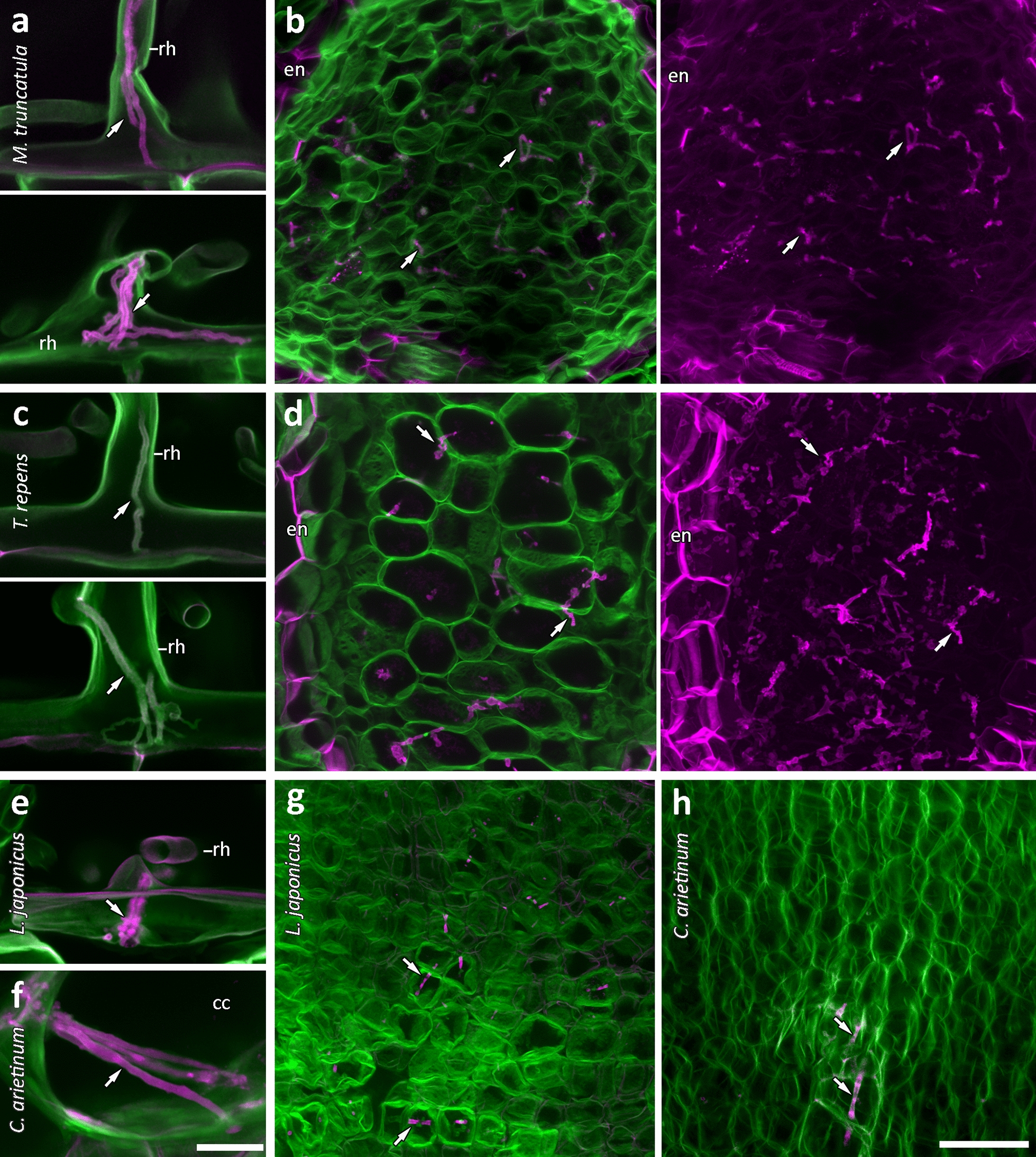
Rhodamine-123-PAS/calcofluor white labelling of infection threads in four legumes. Root hair and nodule infection threads (arrows), respectively, in *M. truncatula* (**a**, **b**), *T. repens* (**c**, **d**), and *L. japonicus* (**e**, **g**). Cortical and nodule infection threads in *C. arietinum* (**f**, **h**). Rhodamine-123 is shown in magenta; calcofluor white is shown in green. **a**, **c**, **e** Infection threads (arrows) in all but *T. repens* root hairs (**c**) are clearly labelled with rhodamine-123. Maximum projections of only the rhodamine-123 channel in much larger z-stacks show extensive infection thread networks in *M. truncatula* and *T. repens* nodules (**b**, **d**). Images are maximum projections from 5 µm and 6.43 µm (**a**), 8 µm and 49.97 µm (**b**), 3.93 µm and 7.14 µm (**c**), 7.14 µm and 176.46 µm (**d**), 14.29 µm (**e**), 37.15 µm (**f**), 11.43 µm (**g**), and 20 µm (**h**) confocal z-stacks. Images **a**–**g** were captured using a 63× objective. Image **h** was captured using a 40× objective. Scale bars equal 25 µm (**a**, **c**, **e**, **f**) and 50 µm (**b**, **d**, **g**, **h**). *rh* root hair, *en* endodermis, *cc* cortical cell. Original resolution versions of images **b** and **d** are available in Additional file 1: Figures S2, S3

**Fig. 5:**
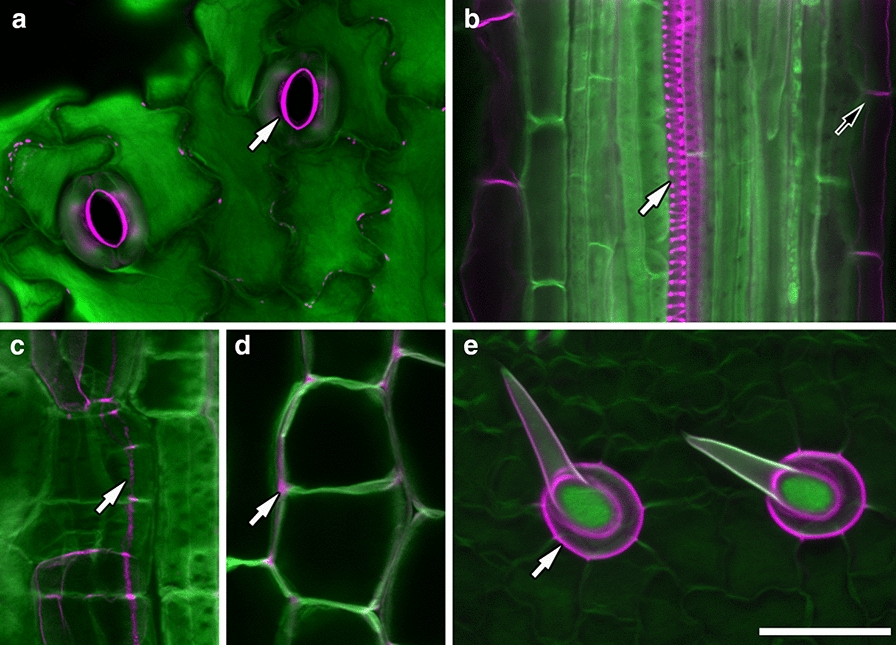
Rhodamine-123-PAS/calcofluor white labelling also differentiates several other tissue types. Stomata thickened inner cell walls (**a**, arrow); xylem vessels (white arrow) and mature endodermis (black arrow) (**b**); casparian strips (**c**, arrow); and non-cellulosic cell wall matrix (**d**, arrow) in *M. truncatula*; and trichomes of *L. japonicus* (**e**, arrow) are labelled with rhodamine-123 (magenta), in contrast to surrounding cell walls labelled with calcofluor white (green). Images **a** and **c**–**e** are maximum projections of 7.14 µm (**a**), 3.57 µm (**c**), 1 µm (**d**), and 30 µm (**e**) confocal z-stacks. Images **a**–**d** were captured using a 63× objective. Image **e** was captured using a 40× objective. Scale bar equals 50 µm

We then tested the effectiveness of this method in infected roots of three other nodulating legumes: *Lotus japonicus*, *Cicer arietinum* (chickpea), and *Trifolium repens* (white clover) (Fig. 4). Root hair infection threads in *Lotus* (Fig. 4e) labelled equivalently to *M. truncatula* (Fig. 4a). However, *T. repens* infection threads did not show the strong rhodamine-123 label (Fig. 4c). Infection threads were also detectable throughout whole cleared nodules in *M. truncatula* (Fig. 3b), *L. japonicus* (Fig. 4g), and *C. arietinum* (Fig. 4h). Here, infection threads of *T. repens* nodules did show strong rhodamine-123 labelling in contrast to calcofluor white labelling of surrounding nodule cell walls (Fig. 4d). It is unclear why *T. repens* infection threads were stained only in nodules. The strong differential labelling of infection threads within *T. repens* and *M. truncatula* nodules also made it possible to show more extensive infection thread networks by creating maximum projections of much larger confocal z-stacks and showing only the rhodamine-123 channel (Fig. 4b, d). Rhodamine-123 staining of nodule infection threads however, was more variable than in root hairs, and more mature nodules lacked rhodamine labelling in most infection threads. The combined rhodamine-123 PAS plus calcofluor white stain also differentiated several other areas including xylem vessels, mature endodermis, Casparian strips, non-cellulosic cell walls, trichomes, and lignified rings in stomata (Fig. 5).

**Fig. 6:**
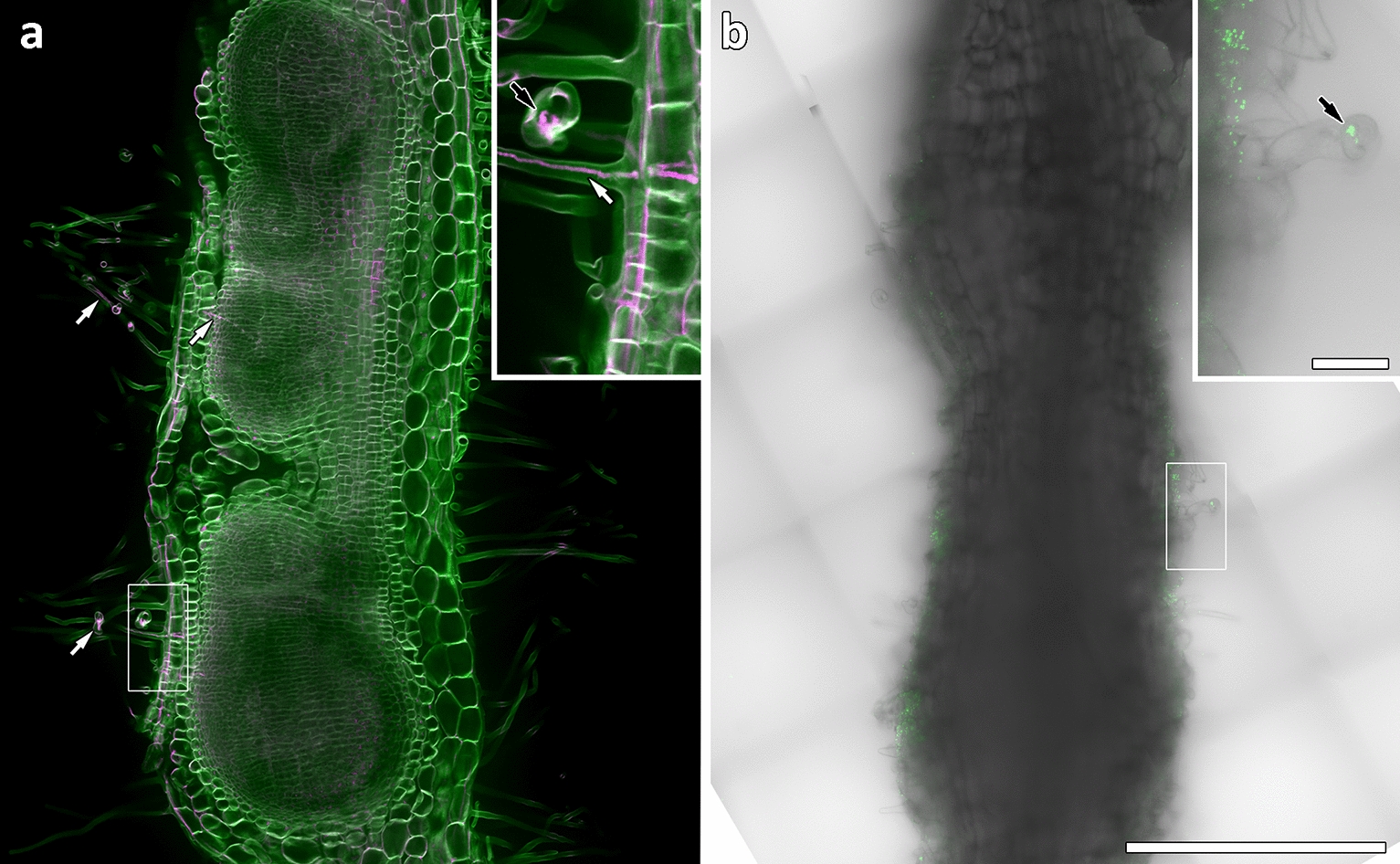
Rhodamine-123-PAS/calcofluor white labelling is more effective than GFP-tagged rhizobia for imaging infection threads in whole roots. Inoculation zones in whole *M. truncatula* roots at equivalent stages of nodule development. **a** Optical section through cleared inoculation zone labelled with rhodamine-123 (magenta) and calcofluor white (green) shows separate nodules; all cell layers; and infection threads (white arrows) and an infection chamber (inset black arrow). **b** An equivalent position in a fresh root inoculated with GFP-tagged rhizobia. Root hairs imaged by DIC are poorly defined and no internal cell layers are visible due to the imaging depth. Inset shows GFP-tagged rhizobia in an infection chamber (arrow). Images are 4 × 6 tile scans captured using a 40× objective. Scale bars equal 500 µm, and 50 µm (inset)

We then compared the imaging of nodules inoculated with GFP-tagged rhizobia with nodules stained using our rhodamine-123 PAS/calcofluor white method. After staining with rhodamine-123 PAS/calcofluor white, an optical section tile-scan through an entire inoculation zone showed that each individual nodule was clearly distinguishable (Fig. 6a). Furthermore, every cell layer was visible to at least 350 µm depth, and infection threads shown in magenta were visible in root hairs and even extending into nodules (Fig. 6a, white arrows). Rhodamine-123 also clearly identified an infection chamber (Fig. 6a inset, black arrow). A root infected with GFP-tagged rhizobia was also imaged at a comparable stage of nodule development (Fig. 6b). GFP fluorescence was imaged in combination with DIC to show both fluorescent bacteria and plant cells. Compared to the cleared and stained sample in Fig. 6a, little internal detail was discernible. More central root tissues could not be resolved because without cytoplasmic clearing the incident laser illumination is absorbed by the first few micrometres of tissue. Hence neither GFP-tagged bacteria nor cell outlines can be identified. Only one infection chamber was detectable and the abundant rhizobia on the surface of the root made it difficult to distinguish bacteria within cells/infection threads. These issues can be partially overcome by imaging infected root hairs closer to the surface of the slide, or in thin sections, but this restricts the information that can be gained when compared to imaging whole inoculated zones.

## Discussion

Although the use of confocal microscopy coupled with cell wall labelling by fluorescent dyes is a common and effective technique for imaging plant cells, it has not been fully utilized for the imaging of nodules and infection threads. Here, we have improved the ability to image infection threads and their structural details in 3D by utilizing confocal microscopy and the intense fluorescent rhodamine-123 PAS plant cell wall stain. Additionally, we developed a method of labelling infection threads differentially from the surrounding cell walls by counterstaining the rhodamine-123 PAS with calcofluor white. Infection threads labelled by the rhodamine-123 PAS method alone were brighter than the surrounding cell walls (Fig. 2), and the optical stability of the PAS label allowed for high resolution imaging. The addition of the calcofluor white counterstain to separate the intense rhodamine-123 label in the infection thread from the surrounding cell walls allowed infection threads in whole infected roots to be easily located; and allowed the full separation of infection threads from surrounding cell walls in 3D reconstructions of infected root hairs.

These techniques in whole infected roots cleared with bleach allowed infection threads to be imaged anywhere within samples, up to a depth of ~ 400 µm. This ability to image these structures throughout whole samples, and in 3D, is an advantage over traditional histochemical techniques such as toluidine blue labelling or X-gluc staining of GUS-tagged proteins, given brightfield microscopy cannot image deep within samples nor generate 3D information. The same advantage applies over the use of GFP-tagged rhizobia to image infection threads, as this is also most often used in uncleared samples and therefore does not allow deep optical sectioning; and given it is the bacteria imaged not the infection thread itself, 3D structural information cannot be gained. It is worth noting that there are clearing methods that retain GFP fluorescence and allow deeper optical sectioning, but these methods take a minimum of 1 week [22] compared to only 1–3 h of bleach clearing in our method. It is also worth noting that, although our method is best used with confocal microscopy, it also benefits the imaging of infection threads with more common epifluorescence microscopes, as demonstrated in Additional file 1: Figure S4.

The reason PAS rhodamine-123 labels infection threads more strongly than surrounding cell walls will require further investigation. However, the layering effect seen in infection threads (Fig. 3), and the contrasting rhodamine-123 labeling of several additional tissue types (Fig. 5) suggests that calcofluor white is either quenching or displacing the rhodamine-123 stain in cellulosic cell walls, leaving rhodamine-123 visible only in tissues not labelled strongly with calcofluor white. Calcofluor white is reported to bind to the β-1, 4-glucan units of cellulose [28] and has been used as a ‘cellulose specific’ dye, although it was more recently shown to be somewhat more promiscuous in its targets [29]. The PAS method, as a general stain for carbohydrates, is known to label hemicellulose, pectins, lignins, and glycoproteins, in addition to labelling cellulose [30–32]. This would account for the retention of rhodamine-123 fluorescence in lignin-rich xylem, endodermis, and trichome cell walls, and in glycoprotein-rich extracellular matrices.

The infection thread matrix is also known to be glycoprotein rich [11, 33], and would therefore be stained by rhodamine-123 and not by calcofluor white. This would suggest the inner rhodamine-123 layer observed in infection threads is the matrix, and the outer layer is the wall of the thread which, as an extension of the root hair cell wall, would be primarily cellulosic. This hypothesis is supported by the cellulose-specific dye, direct red 23 [27], labelling infection threads in combination with rhodamine-123 in the same way as calcofluor white (Additional file 1: Figure S5). If our hypothesis that the infection thread matrix is stained by rhodamine-123 is correct, this technique has potential for use beyond the imaging of infection threads, as little is known of the nature and function of the infection thread matrix. The lack of rhodamine-123 labelling in *T. repens* root hair infection threads (Fig. 4) may indicate a species-specific difference in infection thread matrix composition. Similarly, the variations in infection thread labelling within nodules, and as nodules age, may indicate developmental changes in matrices as infection threads age and bacteria are released. Such variations in infection thread matrices between species, and during infection thread development have not been well described; and besides antibody localisation of matrix glycoproteins by TEM [33], there has been no way of specifically localising and imaging infection thread matrices. We propose that the novel method presented in this paper will enhance the ability to study infection thread matrices, and rhizobial infection more generally.

Given the consistent results of our method in the four legume species tested, it is expected to be applicable to infection thread labelling in other nodulating legumes, with optimisation. Our advice to those trialling the method in species not tested here, is adjustment of the clearing, staining, and oxidation times. Lotus, for example, was oxidised sufficiently by bleach during clearing, and did not require oxidation by periodic acid for best results. In addition to legumes, our method may also be applied for more general component labelling (see Fig. 5) of cell walls in any other plant species.

## Conclusions

The novel combination of rhodamine-123 PAS and calcofluor white stains coupled with clearing of infected tissue and confocal microscopy developed here is an improvement on traditional histochemical and GFP-based techniques in imaging infection thread structure. It is transferable to at least three other nodulating legume species, and with optimisation is expected to be applicable to further species. Its ability to separate distinct components of infection threads may prove valuable in understanding infection thread development; and its power to visualize the 3D structure of infection threads will aid in further research relating to their nature and elongation.

## Methods

### Plant growth conditions

Species used in this study were: *Medicago truncatula* wildtype cultivar Jemalong A17 (South Australian Research and Development Institute, Adelaide, Australia); *Lotus japonicus ecotype* Gifu B-129 (Biological Resource Center In Lotus and Glycine, University of Miyazaki, Japan); *Trifolium repens* cultivar Haifa (Cleanseeds, Bungendore, NSW, Australia); and *Cicer arietinum* cultivar Sonali (Angela Pattison, University of Sydney, NSW, Australia).

*Trifolium repens* and *Lotus japonicus* seeds were scarified with sand paper. *Medicago truncatula* seeds were scarified by a 3-min incubation in concentrated sulphuric acid, followed by five washes with sterile MilliQ water. All seeds were surface sterilised with 6% (w/v) sodium hypochlorite for 5–7 min, or 30 min (*C. arietinum*), followed by five washes and a 2–3 h soak in sterile MilliQ water. Sterile seeds were sown on Fåhraeus (F) medium [34] and incubated at 4 °C, or 25 °C (*C. arietinum*), in the dark for 48 h. To synchronise germination, seeds were then incubated at 25 °C for 24 h (*T. repens*, *M. truncatula*), or 72 h (*L. japonicus*). Germinated *M. truncatula*, *L. japonicus*, and *T. repens* seeds were transferred to F media plates, semi-sealed with Parafilm, and placed vertically in containers with black cardboard in between plates to shield roots from light. *C. arietinum* seedlings were transferred to seed germination pouches (Mega International, Minnesota) and supplied liquid F media. Plants were incubated in growth chamber conditions of a 16-h-light and 8-h-dark period at 150 µmol/m^2^ s light intensity, at 25 °C.

### Rhizobia culture and inoculation

*Sinorhizobium meliloti* strain 1022 (for *M. truncatula*); *Rhizobium leguminosarum* bv*. trifolii* strain ANU843 (for *T. repens*); *Mesorhizobium loti* strain MAFF303099 (for *L. japonicus*); *Mesorhizobium ciceri* Strain CC1192 (for *C. arietinum*); and an *S. meliloti* strain 1021 constitutively expressing gfp [35] for visualizing rhizobia in *M. truncatula*; were maintained at 28 °C on Bergersen’s Modified Medium (BMM) [36]. Before inoculation, bacteria were cultured overnight at 28 °C in liquid BMM (*S. meliloti*), or liquid Tryptone-Yeast (TY) medium (*R. leguminosarum* bv*. trifolii, M. loti, M. ciceri*), then adjusted to an optical density (OD_600 nm_) of 0.05, or 0.1 (*M. ciceri*). Bacterial suspensions were spot-inoculated onto plant roots, by applying a 5 µl drop onto the root nodulation-susceptible zone (~ 2 mm behind the root tip) [37].

### Sample fixation and clearing

Whole nodulating roots, leaves, or cotyledons were incubated at least overnight in 3:1 (v:v) ethanol:acetic anhydride fixative solution. Samples in fixative can be kept at 4 °C for several months without degradation. To clear, fixed samples were first washed in 70% ethanol; then incubated in either 9% (w/v) sodium hypochlorite (*M. truncatula, L. japonicus, C. arietinum*), or 6% (w/v) sodium hypochlorite (*T. repens*) at 28 °C with gentle shaking for 1 h, or until samples appeared translucent. Cleared samples were then washed in ~ 500 ml distilled water with gentle shaking for 30 min.

### Staining

Initial staining of plant tissue was carried out according to the modified Periodic Acid Schiff (PAS) method developed by Rae et al. (2020). Cleared samples were first oxidised for 5 min in 1% (w/v) periodic acid, washed briefly in distilled water, incubated in 2 µg/ml rhodamine-123 in water for 30 min, rinsed briefly in water, then mounted in 100% glycerol. To counterstain with calcofluor white or direct red 23, samples were washed briefly after staining with rhodamine-123, then incubated for 30 min in either 0.5 mg/ml calcofluor white in water, or 0.1 mg/ml direct red 23 diluted in water from 5 mg/ml in 10× PBS [38]; then washed briefly in water (calcofluor white), or 10× PBS (direct red 23) before mounting in 100% glycerol. Thick samples were mounted between two coverslips to allow either side of the sample to be imaged directly by flipping the coverslips. Cover slips were separated by a thin layer of high vacuum grease to prevent sample crushing, and placed on microscope slides with a small drop of grease under opposite corners to lightly adhere coverslips. Periodic acid, rhodamine-123, calcofluor white, and direct red 23 were supplied by Sigma Chemicals.

### Microscopy

Stained plant samples were imaged on a Leica SP8 confocal microscope (Leica Microsystems) with either a 63 × 1.2NA water-immersion objective, a 40 × 1.1NA water-immersion objective, or a 40 × 0.6NA dry objective. Rhodamine-123 and Calcofluor white stains were excited simultaneously with 514 nm and 405 nm diode argon lasers, respectively, with fluorescence emission at 520–620 nm and 410–510 nm, respectively. Pixel resolution was set to 1024 × 1024. Offset level was set at zero, pinhole size was set at 133 μm, or 117 µm (Fig. 1), and gain level was set for optimum exposure time. Z-stacks for 3D reconstruction were recorded at 0.35 µm intervals.

For epifluorescence imaging, stained plant samples were imaged on a Leica DM5500 compound microscope with a 100 × 1.4NA oil-immersion objective. 360/40 and 500/20 band pass filters excitation filters with 470/40 and 535/30 band pass suppression filters were used for calcofluor white and rhodamine-123 stains, respectively.

### Image analysis

All post acquisition image processing was carried out in the Leica application suite X (LasX, Leica Microsystems) software, and figures were produced with Adobe Photoshop. The LasX 3D visualisation module was used to create Z-stack 3D reconstructions and movies.

## Supplementary Information

**Additional file 1: Figure S1.** Medicago truncatula infection threads stained with neutral red, ruthenium red, safranin-O, and calcofluor white. **Figure S2.** Original resolution version of Fig. 4b, **Figure S3.** Original resolution version of Fig. 4d. **Figure S4.** Epifluorescence images of stained infection threads in Medicago truncatula. **Figure S5.** Comparison of Medicago truncatula infection threads labelled with rhodamine-123 PAS (rh123) counterstained with calcofluor white (cw), and rhodamine-123 counterstained with direct red 23 (dr23).

**Additional file 2: Movie S1.** 3D reconstruction from a confocal z-stack of infection threads within a M. truncatula root hair stained with rhodamine-123 (magenta) and counterstained with calcofluor white (green).

**Additional file 3: Movie S2.** 3D reconstruction from a confocal z-stack of an infection thread within a *M. truncatula* root hair stained with rhodamine-123 (magenta) and counterstained with calcofluor white (green).

**Additional file 4: Movie S3.** 3D reconstruction from a confocal z-stack of infection threads within *M. truncatula* root hairs stained with rhodamine-123 (magenta) and counterstained with calcofluor white (green).

## Data Availability

All data generated or analysed during this study are included in this published article and its additional information files.

## Abbreviation

PAS: Periodic Acid Schiff
