# Supplementary material for: New methods for confocal imaging of infection threads in crop and model legumes
Source: Plant Methods. 2021 Mar 7;17:24. doi: 10.1186/s13007-021-00725-6 (PMC7938587; doi:10.1186/s13007-021-00725-6)
Supplement: Supplementary file 1 — Additional file 1: Figure S1. Medicago truncatula infection threads stained with neutral red, ruthenium red, safranin-O, and calcofluor white. Figure S2. Original resolution version of Fig. 4b, Figure S3. Original resolution version of Fig. 4d. Figure S4. Epifluorescence images of stained infection threads in Medicago truncatula. Figure S5. Comparison of Medicago truncatula infection threads labelled with rhodamine-123 PAS (rh123) counterstained with calcofluor white (cw), and rhodamine-123 counterstained with direct red 23 (dr23). [file 13007_2021_725_MOESM1_ESM.docx]

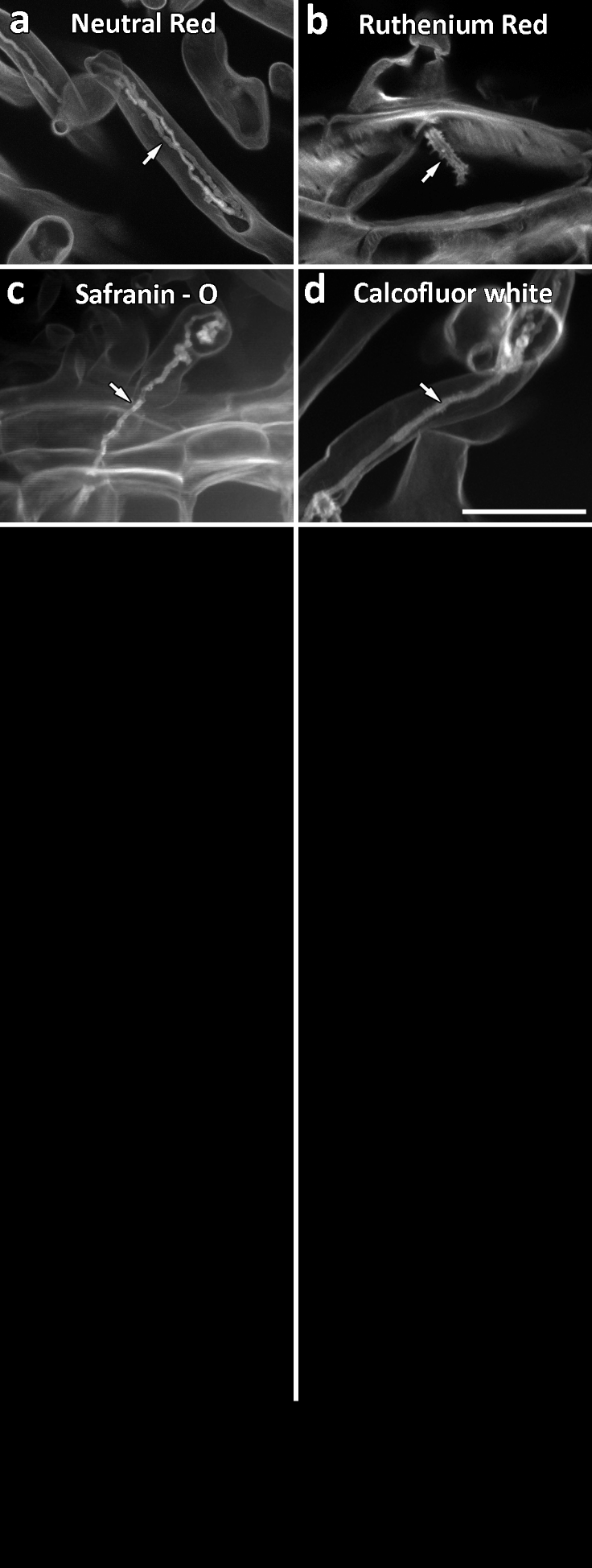


**Figure S1.** *Medicago truncatula* infection threads stained for 30 min with **(a)** 0.1 mg/ml neutral red, **(b)** 0.1 mg/ml ruthenium red, **(c)** 0.1 mg/ml safranin-O, or **(d)** 1 mg/ml calcofluor white. Images **a** & **b** are maximum projections of 10.72 µm and 4.64 µm confocal z-stacks, captured using a 63x objectives. Images **c** & **d** were captured using a 40x objective. Scale bar = 50 µm.


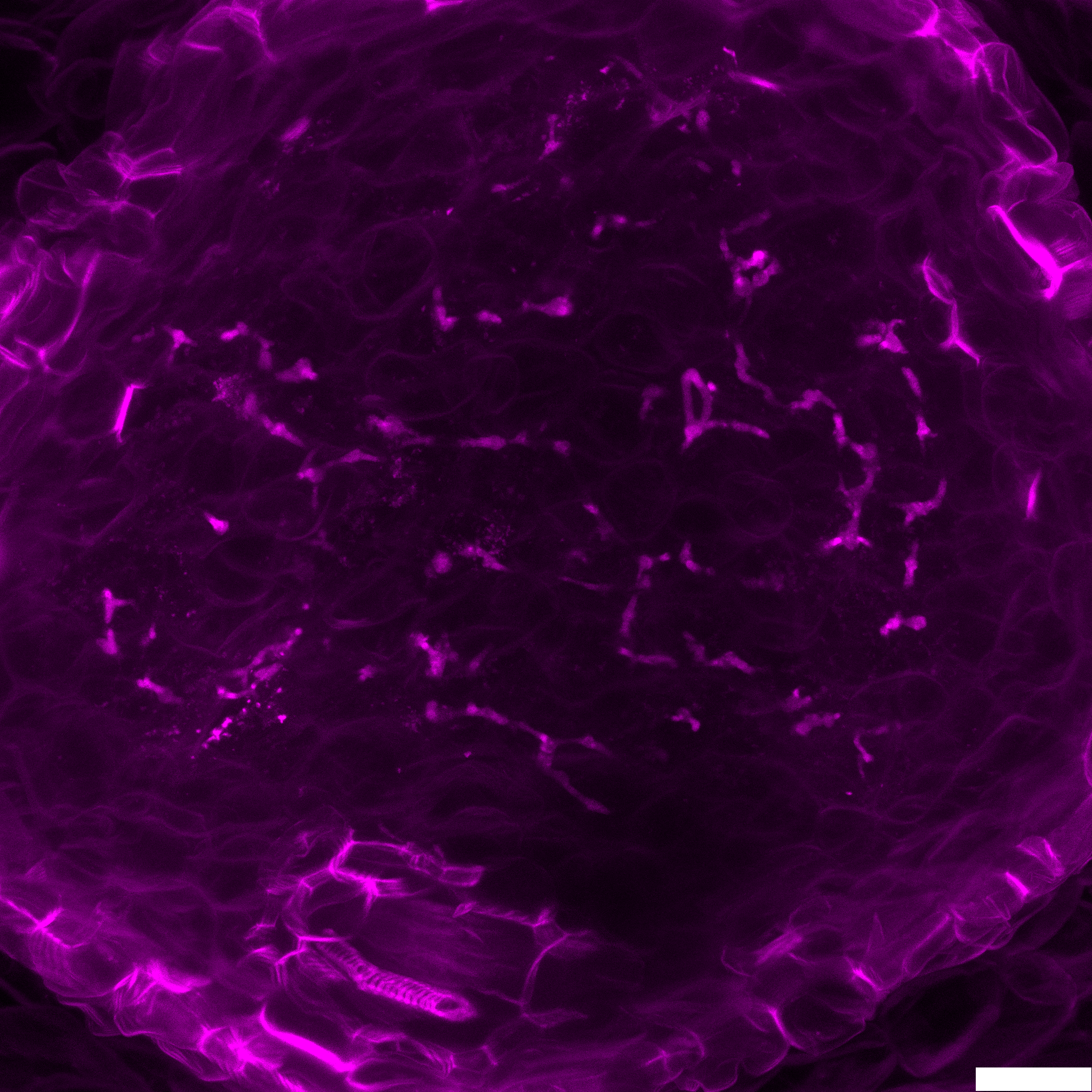


**Figure S2.** Original resolution version of Figure 4b. Scale bar = 25 µm.

**
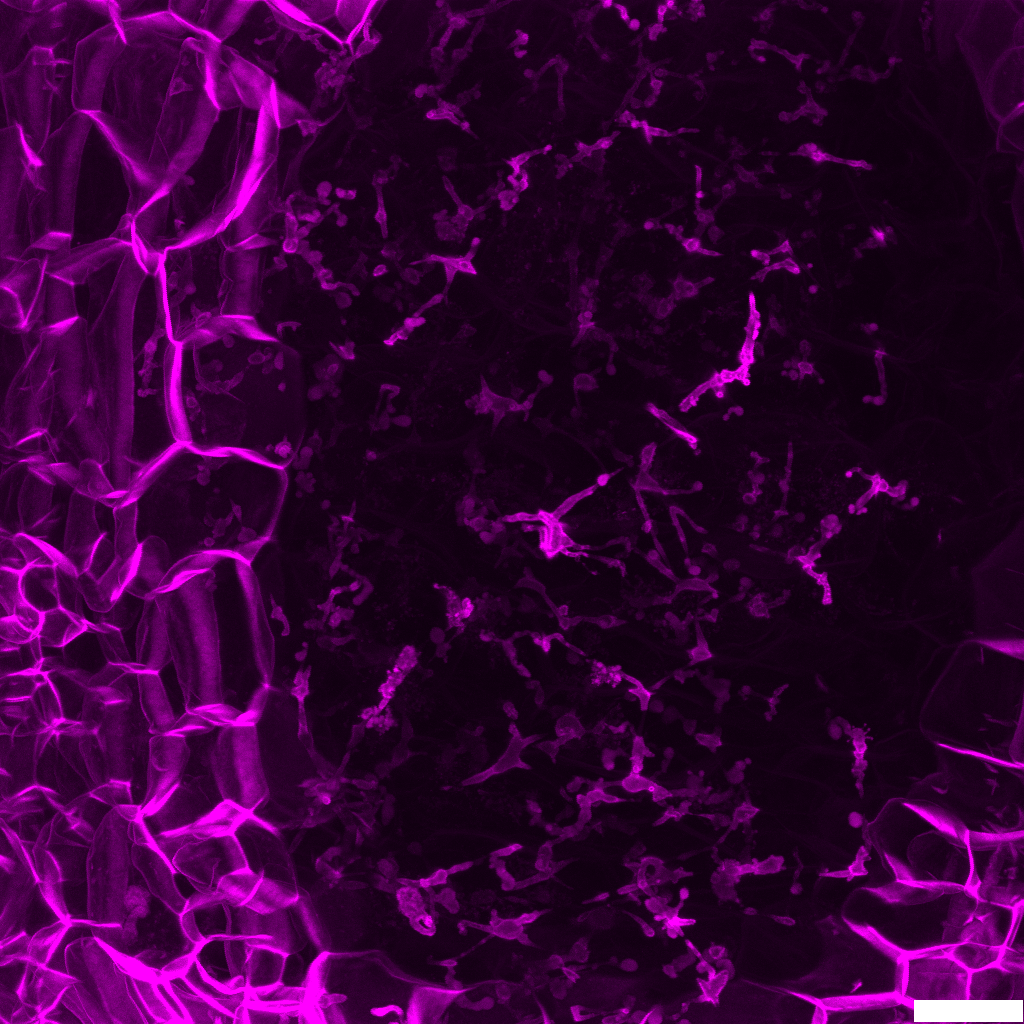
**

**Figure S2.** Original resolution version of Figure 4d. Scale bar = 25 µm.


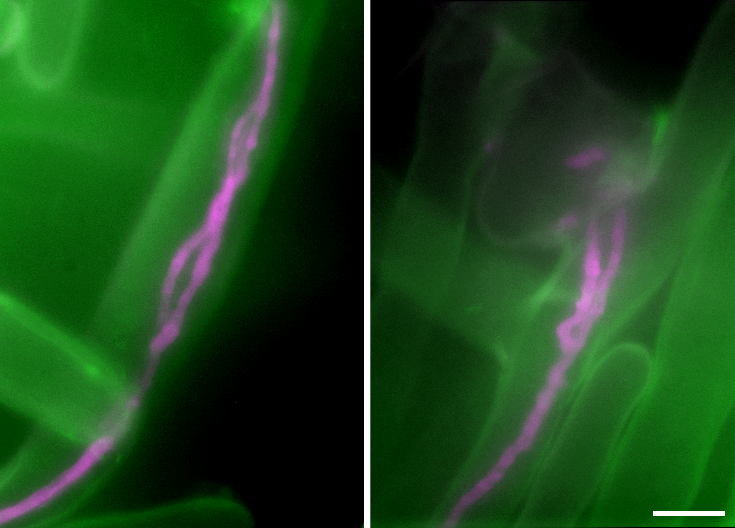


**Figure S4.** Epifluorescence images of stained infection threads in *Medicago truncatula*. *M. truncatula* root hairs stained with PAS rhodamine-123 (magenta) then with calcofluor white (green). Images acquired on a Leica DM5500 compound microscope using a 100x objective. Scale bar = 10 µm.


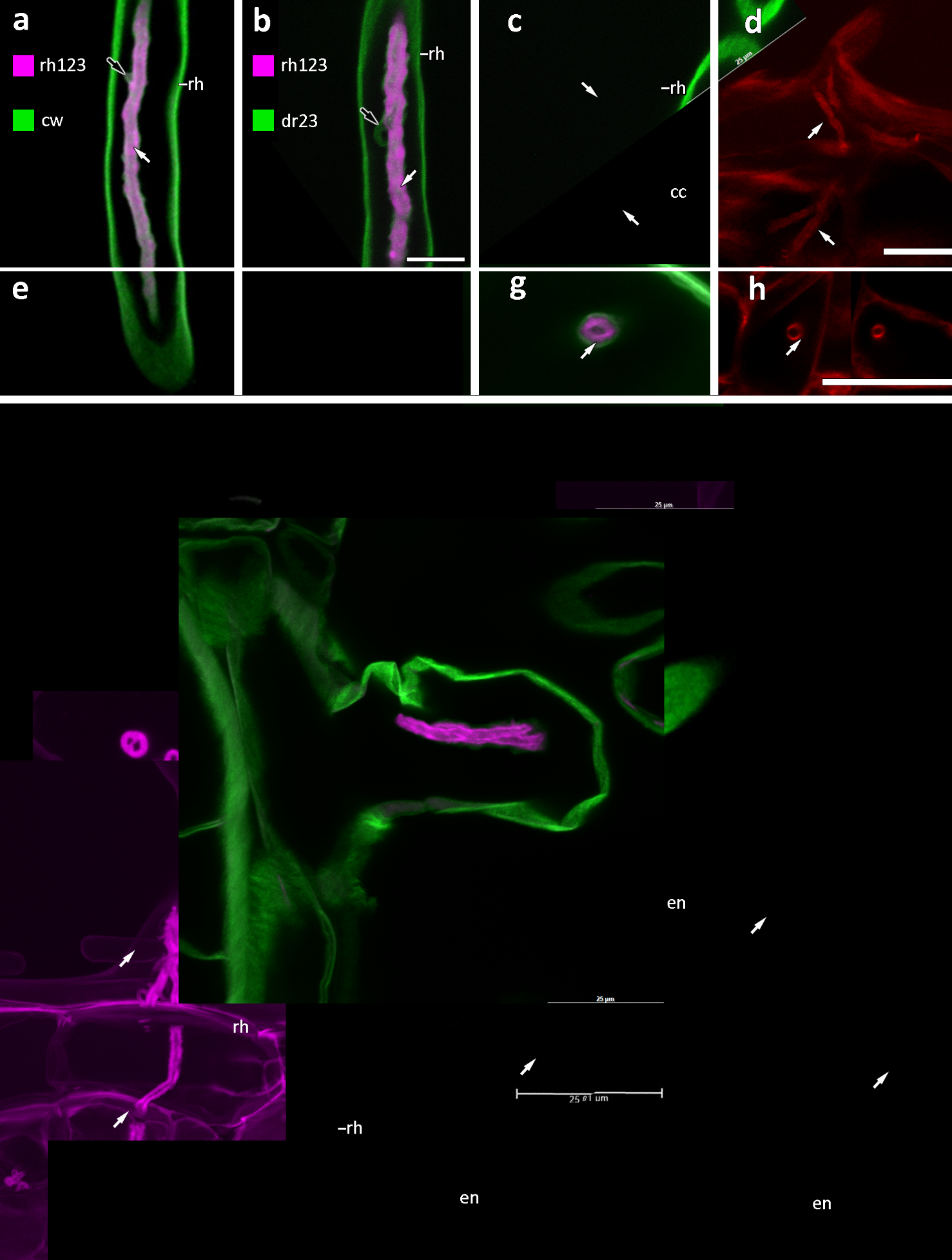


**Figure S5.** Comparison of *Medicago truncatula* infection threads labelled with rhodamine-123 PAS (rh123) counterstained with calcofluor white (cw) **(a)**, and rhodamine-123 counterstained with direct red 23 (dr23) **(d)**. Rhodamine-123 counterstained with calcofluor, and direct red 23, appear identical in their labelling of infection threads. Direct red 23 labels the root hair cell walls and outer infection thread components (black arrows) in the same way as calcofluor white; against the stronger rhodamine-123 label in the body of infection threads (white arrows). Images are maximum projections of 2.14 µm confocal z-stacks, captured using a 63x objective. Scale bar = 10 μm. rh = root hair.
